# Supplementary material for: Saccharomyces boulardii (CNCM I-745) improves intestinal damage in sepsis by remodeling intestinal flora structure
Source: Front Cell Infect Microbiol. 2025 Jul 31;15:1602792. doi: 10.3389/fcimb.2025.1602792 (PMC12350329; doi:10.3389/fcimb.2025.1602792)
Supplement: Supplementary file 1 [file DataSheet1.zip › summary/summary/6_Taxonomy_community/1_Taxonomy_profiling/3_Class/Group/Class_abund_top30.Group.cluster.pdf]

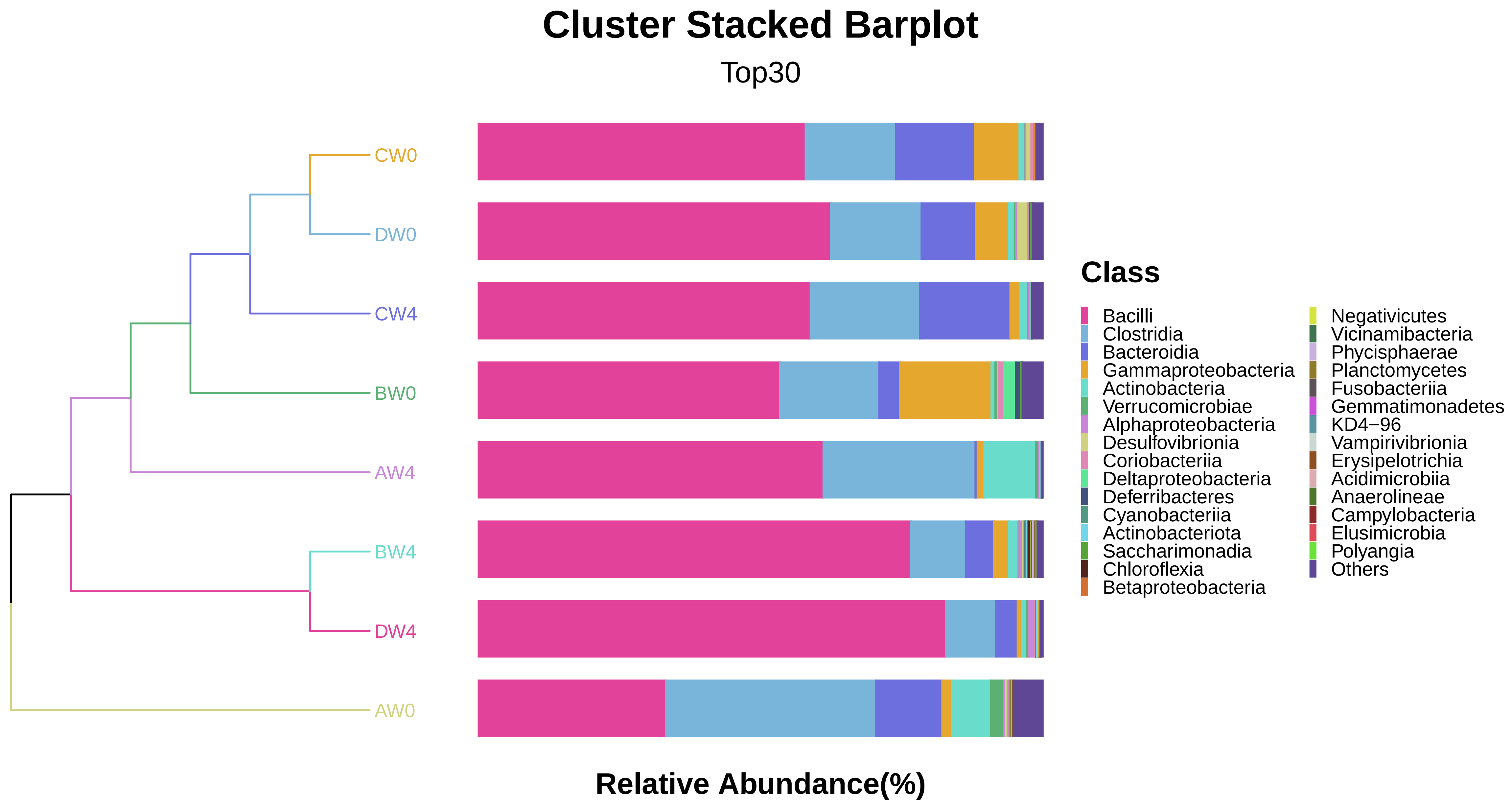

Cluster Stacked Barplot
Top30
CW0
DW0
CW4
BW0
AW4
BW4
DW4
AW0
Relative Abundance(%)
Class
Bacilli
Clostridia
Bacteroidia
Gammaproteobacteria
Actinobacteria
Verrucomicrobiae
Alphaproteobacteria
Desulfovibrionia
Coriobacteriia
Deltaproteobacteria
Deferribacteres
Cyanobacteriia
Actinobacteriota
Saccharimonadia
Chloroflexia
Betaproteobacteria
Negativicutes
Vicinamibacteria
Phycisphaerae
Planctomycetes
Fusobacteriia
Gemmatimonadetes
KD4−96
Vampirivibrionia
Erysipelotrichia
Acidimicrobiia
Anaerolineae
Campylobacteria
Elusimicrobia
Polyangia
Others
